# Nitrogen-Doped Multi-Scale Porous Carbon for High Voltage Aqueous Supercapacitors

**DOI:** 10.3389/fchem.2018.00475

**Published:** 2018-10-17

**Authors:** Xichuan Liu, Rui Mi, Lei Yuan, Fan Yang, Zhibing Fu, Chaoyang Wang, Yongjian Tang

**Affiliations:** ^1^Shanghai EBIT Lab, Key Laboratory of Nuclear Physics and Ion-beam Application, Department of Nuclear Science and Technology, Institute of Modern Physics, Fudan University, Shanghai, China; ^2^Science and Technology on Plasma Physics Laboratory, Research Centre of Laser Fusion, China Academy of Engineering Physics, Mianyang, China

**Keywords:** supercapacitors, aqueous electrolyte, water in salt, porous carbon, high voltage, energy density

## Abstract

Recently, “Water-in-salt” electrolyte has been reported to extend the working voltage of aqueous supercapacitor. However, this electrolyte needs the electrode materials possess some good features such as proper pore structure, high electron and ion conductivity. Herein, we fabricated the nitrogen-doped multi-scale porous carbon (NMC) by the simple enriching melamine-resorcinol-formaldehyde xerogels method with integrating triblock copolymer for micro-pores formation. All the results confirmed that our NMC is provided with a very high specific surface area (3,170 m^2^ g^−1^) and its monoliths are composed of multi-scale porous structure. By employing the nanostructured NMC as electrode materials, we have investigated the capability for high-voltage aqueous supercapacitor applications. The superconcentrated “Water-in-salt” electrolyte expand stability operating potential window of aqueous symmetric supercapacitor up to 2.4 V with a high energy density of 33 Wh kg^−1^ at power density of 0.3 kW kg^−1^. Our studies indicate that the NMC is potential materials for high performance over wider voltage range.

## Introduction

For the fast consumption of fossil fuels leads to global severe environmental issues and energy crisis, exploitation of new energy sources become an urgent issue for humanity. For decades, many works have been devoted to develop new technologies to use new energy sources from the ambient or renewable sources like wind, solar, tide, electromagnetic fields, mechanical movement and so on, and converted to electrical energy in an energy storage device like batteries (Zhao et al., 2017; Zhang et al., 2018a). However, due to the intermittent nature of these energy sources, batteries will be charged repeatedly which resulted in rapid decay of their cycle life. In this case, supercapacitors (SCs) with favorable features of long cycling stability, fast charging/discharging ability and high power density are generally more suitable than batteries (Jia et al., 2018; Liu et al., 2018b; Qu et al., 2018). Specifically, since the SCs have high specific power characteristic, it also have been widely used in a wide variety of applications such as portable electronics, electric or hybrid electric vehicles, aircraft and smart grids.

Nevertheless, the low energy densities of SCs restrict its widespread applications. According to the Equation (1), the energy density (E) is related to the capacitance (C) and operating voltage (V). For increasing the energy stored in SCs, previous works have been widely focused on the improvement of capacitance which takes advantage of various topical subjects like the selection, the construction, and the modification of electrode materials (Zhong et al., 2015; Dai et al., 2017; Liu et al., 2018a). So far, few researches focus on the crucial factors correlating to the operating voltage, even though it is more efficient to increase the energy density and power density (P), according to the Equation (2) (where R is the internal resistance) by expanding the operating voltage, since energy density and power density are directly proportional to the square of voltage.

(1) $$\begin{array}{r} \text{E}=\text{C}{\text{V}}^{2}/2 \end{array}$$

(2) $$\begin{array}{r} \text{P}={\text{V}}^{2}/4\text{R} \end{array}$$

The SCs are usually use three types electrolyte (Zhong et al., 2015): aqueous, organic and ionic liquid (Kühnel and Balducci, 2014). Using organic or ionic liquid electrolyte can efficiently expand the potential window, which because of the organic or ionic liquid electrolyte has a good electrochemical stability with higher decomposition voltage (2.5–4.5 V) than aqueous. However, a series of undesired features severely limit the wide application of organic electrolyte. For example, the SCs with organic and ionic liquid electrolytes often suffer from low capacitances and power densities due to their large-size ion and low ionic conductivity nature. In addition, the organic and ionic liquid electrolytes are not only noxious and flammable result in environmental and security issues, but also require rigorous manufacturing procedures. On the contrary, aqueous electrolytes get more attention due to its inherently safety, low-cost, and easy operation characters. For the smaller-size ion and faster ionic conductivity enable aqueous SCs with larger capacitances and higher power densities (Zao et al., 2016; Hwang et al., 2017; Zeng et al., 2018a). So, it is urgent to study aqueous SCs with both high energy and power density fulfilling the application of the SCs.

The most challenge to obtain high-voltage aqueous SCs is expanding the electrochemical stability window of water (1.23 V), previous studies have devoted to asymmetry structure or neutral aqueous electrolytes, and the highest potential window even beyond 2 V (Yang et al., 2017; Zuo et al., 2017; Fu et al., 2018). Very recently, Yu et al. (2017) summarized the new insight into the high voltage of aqueous SCs. Many crucial tactics of expanding the operating voltage have been studied. Specifically, Xu et al. (Suo et al., 2015) reported an intriguing breakthrough that a superconcentrated lithium bis(trifluoromethane sulfonyl)imide (LiTFSI) aqueous solution named “water-in-salt” electrolyte displays a obviously high electrochemical stability up to 3 V in lithium-ion batteries applications. Obviously, this “water-in-salt” electrolyte also can be used in high voltage aqueous SCs (Gambou-Bosca and Bélanger, 2016; Díez et al., 2017; Reber et al., 2017). For instance, Hasegawa, et al. (Zhao et al., 2016) fabricates symmetric SCs using 5 M LiTFSI aqueous solution achieved a maximum stable operating voltage of 2.4 V. Nevertheless, although using the LiTFSI aqueous solution expand the operating voltage of SCs, it is also suffered the sacrifice of capacitances, which result in the limited increasing of energy density. Therefore, it is needed to choose one proper electrode materials for matching the LiTFSI molecule to maximization the capacitances as well as expanding the operating voltage. In this case, multi-scale carbonaceous materials with high good thermal and chemical stability, good porous network, and satisfactory electrical conductivity have been widely studied, and it is very suitable for SCs. (Huang et al., 2012; Fang et al., 2013; Hasegawa et al., 2016). Moreover, N-doped carbon materials have many attractive functional properties. It also gives more active sites for electrochemical reactions in double layer capacitors (Geng et al., 2011; Ci et al., 2012; Zhong et al., 2013; Zhang et al., 2018b). Furthermore, for the excellent performance of N-doped carbon, it has been studied in long-term performance in SCs (Wen et al., 2012; Zhu et al., 2016; Zeng et al., 2018b).

In this work, Nitrogen-doped multi-scale carbon (NMC) was fabricated by simple sol-gel reaction with additional CO_2_ activation. The structure of this material is comprised of a multi-scaled pore with nano-porous carbon in a network of micron-size percolated hollow-duct. In particular, the prepared NMC was utilized as electrode materials to explore high-voltage aqueous supercapacitors.

## Experimental

### Materials synthesis

NMC were fabricated by sol-gel method from a solution containing resorcinol (R), formaldehyde (F) and melamine (M), followed by aging, solvent exchange, drying and pyrolysis. In a typical process, firstly, 1.6 g of triblock copolymer Pluronic F68 (PEO_76_-PPO_29_-PEO_76_) was dissolved in a small amount of deionized water and ethanol at 60°C, followed by adding 7.7 g of melamine and 18 mL of formaldehyde solution (37 wt %), with vigorous stirring until melamine was completely dissolved. Then, 6.5 g of resorcinol and 8.7 mL of formaldehyde solution were added into the above mixture solution, and stirring until resorcinol was completely dissolved. After that, 6 mL of NaOH solution (0.02 M) was added into the above mixture using as catalyst. At last, amount of deinoized water was added to meet the volume at 50 mL. The mixture was stirred at 60°C for 3 min. Hereafter, the mixture was placed in a sealed container and kept at 60°C for 72 h to finish the gel process. The M-R-F hydrogels containing PEO_76_-PPO_29_-PEO_76_ were immersed in a solution of trifluoroacetic acid and ethanol (3:97 in volume) at room temperature for 72 h. Afterwards, the residual solvent was substitute for ethanol for 6 times per 24 h to remove water. Subsequently, the hydrogels were dried at 60°C and carbonized with argon gas flow rate of 100 mL min^−1^ at 800°C for 4 h. And this nitrogen-containing porous carbon without CO_2_ activation was denoted as NC. In the CO_2_ activation process, the NC was heated in a tubular furnace at 950°Cfor 8 h under a stable CO_2_ flow (150 mL min^−1^) and then the NMC was obtained. For comparison, the commercialized active carbon YP-50 (AC) was purchased from Kuraray chemical co. (Japanese).

### Characterization of the samples

The morphology of the NC and NMC was observed by scanning electron microscopy (SEM) and high-resolution transmission electron microscopy (HRTEM). The crystallographical information and phase of the samples were investigated by X-ray powder diffraction (XRD) and Raman spectroscopy. N_2_ adsorption/desorption isotherms were tested by an AUTOSORB-IQ surface area analyzer (Quantachrome Instrument Corporation) at 77 K. The chemical composition of the NMC was conducted with X-ray photoelectron spectroscopy (XPS).

### Electrochemical measurements

For electrochemical experiments, the working electrodes were fabricated with 80 wt% active materials, 10 wt% acetylene black and 10 wt% polytetrafluoroethylene (PTFE) in ethanol to form a mixture solution. And the mixture was pressed onto stainless steel network at 20 MPa with a diameter of 16 mm and a mass of around 2 mg. The electrode was tested in two- and three-electrode cells. The 20 mol kg^−1^ (m) LiTFSI aqueous solution was used as electrolytes. Two-electrode capacitor was tested in a CR2032-type coin cell with comparable mass of active materials, while a piece of sulfonated polypropylene membrane was employed as separator. In a three electrode cell, a pair of electrodes was used as working and counter electrode respectively, and the saturated calomel electrode (SCE) was used as reference electrode. The galvanostatic charge/discharge tests (GCD) and cycling performance were tested at LAND instrument (CT2001, China). Cyclic voltammetry (CV) was tested in the same range by a CHI 760 electrochemical workstation. Electrochemical impedance spectroscopy (EIS) measurements were performed from 100 kHz to 10 mHz.

## Results and discussion

### Morphologies and crystallographical information

**Figure 1:**
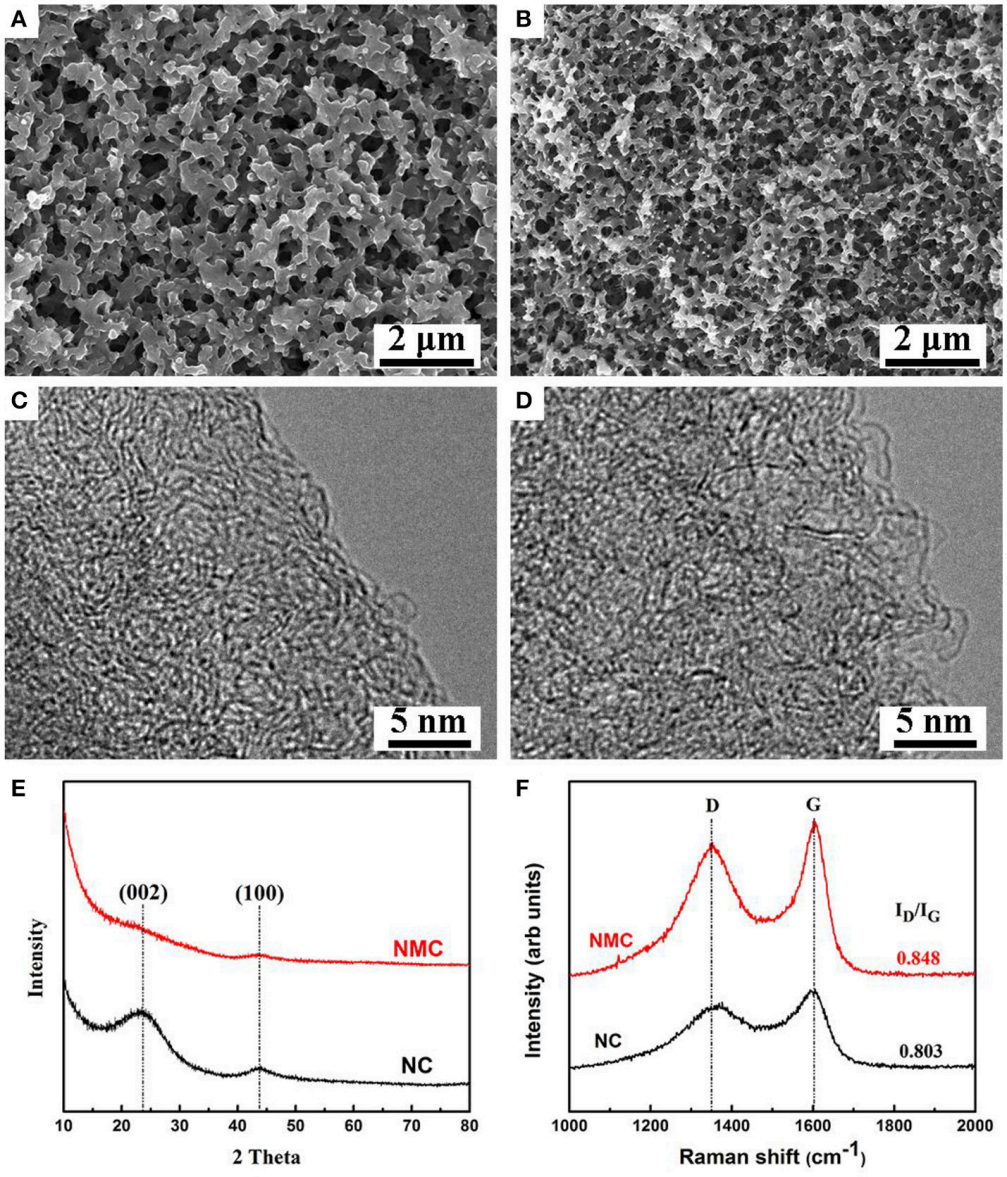
SEM images **(A,B)**, HRTEM images **(C,D)**, XRD spectra **(E)**, and Raman spectra **(F)** of the NC and NMC.

Studies on the R-F sol-gel reaction (Al-Muhtaseb and Ritter, 2003), it is known that the resorcinol is react with water to form hudroxymethyl derivatives (-CH_2_OH), and a condensation reaction of the hudroxymethyl derivatives with F to form methylene (-CH_2_-) and methylene ether (-CH_2_OCH_2_-) bridged compounds. At the same ambient aqueous condition, a sol-gel reaction between M and F were also observed (Raymundo-Pinero et al., 2002). In this work, we simultaneously put the M, R, F, and PEO_76_-PPO_29_-PEO_76_ in one react system, condensation reaction happens among the hydroxymethyl groups between M, R, and F to form small M-R-F clusters, which act as nucleation sites and incessantly increase to form a larger colloids. In this procedure, PEO_76_-PPO_29_-PEO_76_ micelles are coinstantaneous embedded within the growing colloids, which can stabilize the M–R–F three dimensional structure. Figure 1A shows the morphological and structural analysis of NC. It shows that the NC with a three dimensional structure comprises interconnected carbon spheres, and contain pores about one micron in size derived from the decomposition of M-F and PEO_76_-PPO_29_-PEO_76_ (Xu et al., 2012). The NC shows a foam-like microstructure with many internal interconnected channels, and this result is well agreed with other reports (Goldmints et al., 1997; Gutiérrez et al., 2009; Zhou et al., 2013). After activation at 950°C for 10 h by CO_2_ (Figure 1B), the NMC also present a foam-like structure except that the carbon skeleton becomes smaller and the size of primary carbon spheres decreases (Lin and Ritter, 2000). This result is owing to CO_2_ etching effect, so the activation process lead to carbon loss. In addition, one would expect that amount of nano-pores are produced in the carbon skeleton by the CO_2_ etching effect, which could be further measured by N_2_ adsorption-desorption test in detail.

It is known that the CO_2_ activation can produce nano-pores in carbon materials (Lin and Ritter, 2000; Chang et al., 2013) and cause different structure formation in nature, however, these features are hardly detected by SEM images (Figure 1B). So we further adopt HRTEM imaging, XRD patterns and Raman spectra to examine these changes of the prepared carbon materials. Figures 1C,D shows the HRTEM imaging of NC and NMC. It reveals the structure of NC and NMC is basically amorphous in nature. Specifically, some partly graphitized carbon structure can be observed from HRTEM imagines, and these structures have been reduced after CO_2_ activation. This phenomenon is coinciding with the XRD test results shown in Figure 1E. It shows two broad characteristic diffraction peaks at 2θ of 23.5° (002) and 43.6° (101), respectively, which can be regarded as a partly graphitized carbon. On the other hand, XRD pattern of the activated NMC displays a broader peak than the NC due to the decrease of the partly graphitized carbon structure by the CO_2_ etching. Moreover, Raman spectra of NC and NMC (Figure 1F) show two typically broad peaks of D band (around 1350 cm^−1^) and G band (around 1600 cm^−1^). Generally, The D band is related to disordered features of graphitic carbon, while the G band is the typical characteristics of graphitic crystallites (Ji and Zhang, 2009). This result is corresponding to previous reports (Ferrari, 2007; Kicinski et al., 2013; Yi et al., 2017). The intensity ratio of D band and G band (I_D_/I_G_) is the parameter usually used to analyze defective structures in carbonaceous materials. Calculated from the Figure 1F, the I_D_/I_G_ value of NMC and NC is 0.848 and 0.803, respectively, the increased value of the NMC indicating that during the CO_2_ activation, the overall structure comprises graphite grains with an increasing amount of structural defects formed. The result well coincide with the HRTEM and XRD results which reveal the existence of amorphous carbon structure of NMC and NC.

### Textural properties

**Figure 2:**
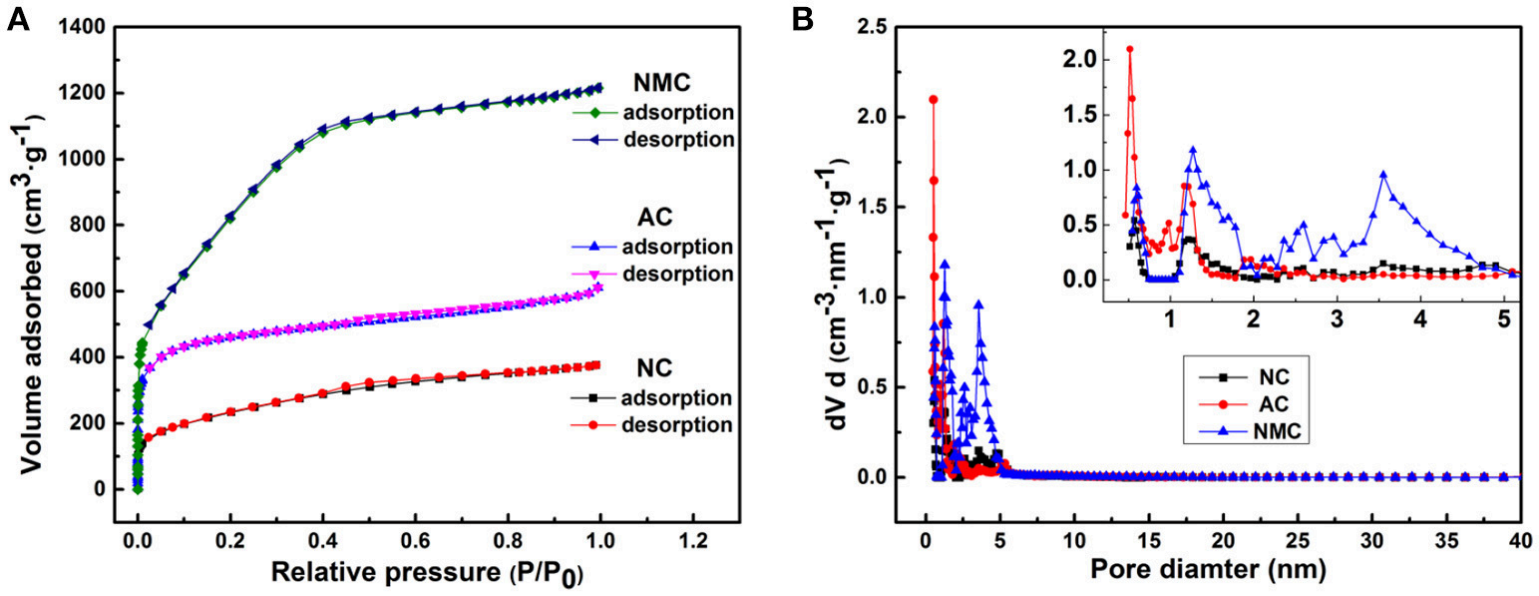
Nitrogen adsorption/desorption isotherms **(A)** and pore size distribution **(B)** of the NC, AC, and NMC.

One expected that our resulted NMC is a multi-scaled carbon material including macropores (>50 nm), mesopores (2–50 nm), and micropores (<2 nm). The nitrogen adsorption/desorption isotherms can examine in detail these pore properties shown in Figure 2A. And the related pore size distributions profiles determined by the density functional theory (DFT) program are shown in Figure 2B. Corresponding pore properties are displayed in Table 1. Obviously, both NMC and AC have a higher absorbed amount than the NC, which reveals that the NMC and AC possess higher specific surface area and pore volume than NC (Table 1). In depth, all these isotherms of NC, NMC and AC possess adsorbed amount at low relative pressure which exhibit Type I characteristics behavior designating the existence of micropores. And the distinct increasing of adsorbed amount for the isotherms can be found, which reveals the increasing amounts of micropores. Another adsorption occurred at middle relative pressure display Type IV characteristics demonstrating the existence of mesopores (Long et al., 2008; Wang et al., 2008). Although, their adsorption curves are mostly consistent with their desorption curves, but the differences also exists at mid-/high-relative pressure. These kinds of isotherms are Type IV curves and Type H4 hysteresis loops, suggesting the existence of slit-shaped pores. This result can be further confirmed by the pore size distributions displayed in Figure 2B. The additional CO_2_ activation process bring abundant 2 nm-pores of the NMC, and it possess a high BET specific surface area of 3,170 m^2^ g^−1^ and a total pore volume of 1.880 cm^3^ g^−1^ shown in Table 1. In summary, NMC possessing high surface area and multi-scale porous structure was successfully prepared. More mesopores exist in NMC (Figure 2B) are beneficial to electrochemical performance in the high-voltage aqueous supercapacitors (Hasegawa et al., 2016).

**Table 1:** Corresponding pore parameters of the NC, AC, and NMC.

	**BET specific surface area (m^2^ g^−1^)**	**Average pore size (nm)**	**Total pore volume (cm^3^ g^−1^)**
NC	840	2.77	0.582
AC	1724	2.20	0.948
NMC	3170	2.37	1.880

### XPS study

**Figure 3:**
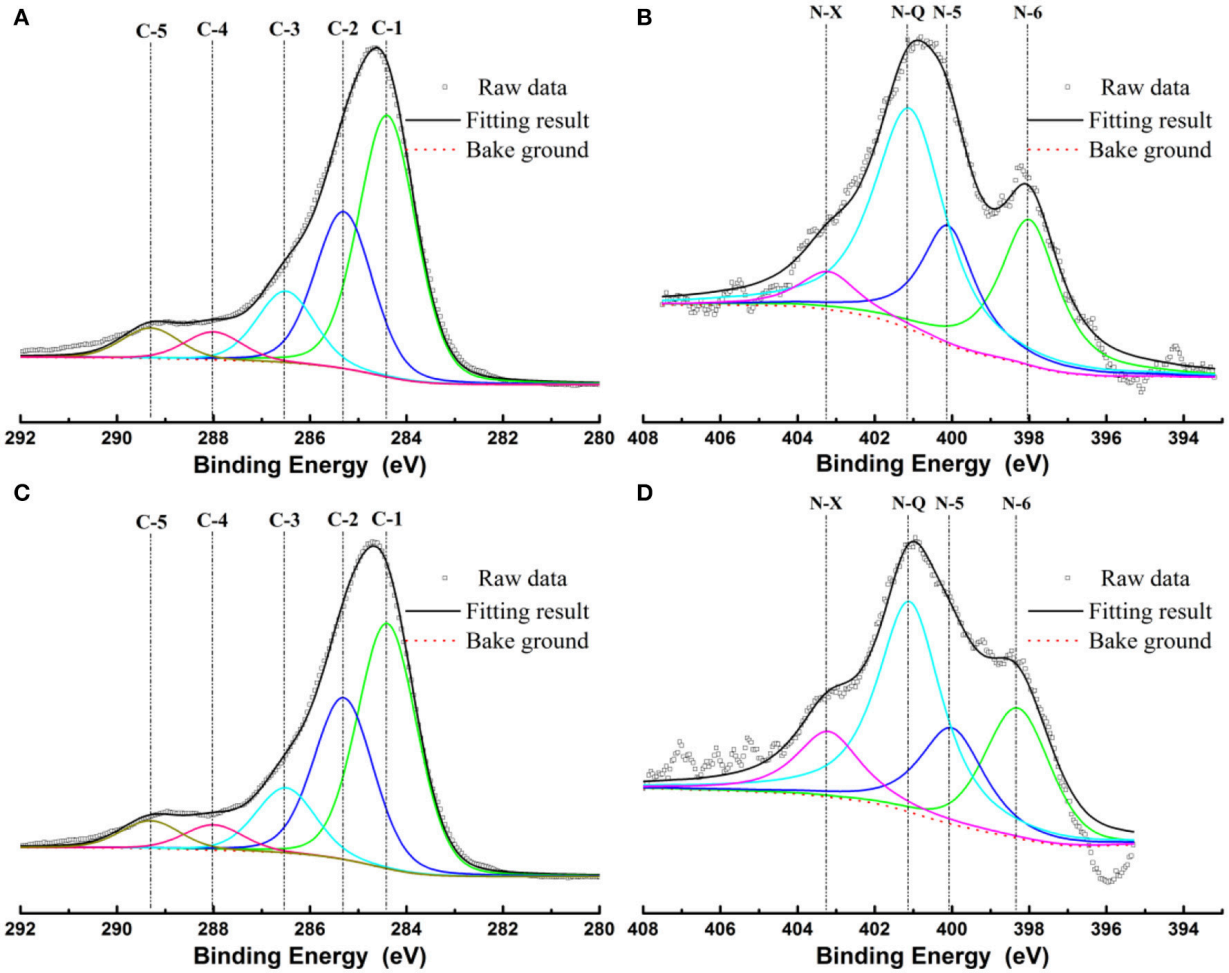
XPS spectra C1s **(A,C)** and N1s **(B,D)** of the NC and NMC, respectively.

**Figure 4:**
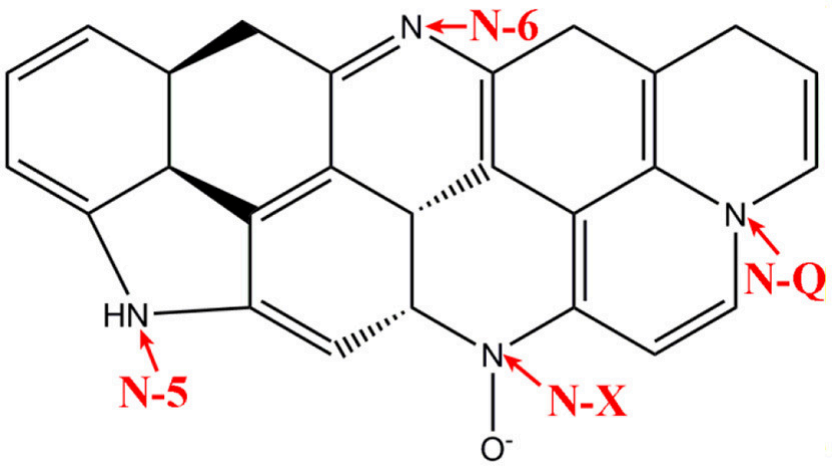
Schematic illustration of different types of nitrogen forms in NMC.

XPS was employed to evaluate the surface chemistry in NC and NMC. As shown in Figure S1, three elements (C, N, O) exist in both NC and NMC. The total nitrogen heteroatom doping content was 4.9 at.% in NC and decreased to 3.2 at.% in NMC. The decrease of nitrogen content is owing to its higher reaction activities than that of carbon during high temperature activation process (Liu et al., 2012). The high-resolution spectrum of C1s and N1s are shown in Figure 3. The C1s spectrum of NC and NMC can be fitted to five peaks show in Figures 3A,C, respectively. The peak located around 284.4, 285.3, 286.5, and 288, 289.3 eV are attribute to the C–C or C = C band (C-1), sp^3^-like defects (C-2), C–N or C–O species (C-3), C = O band (C-4), and π-π^*^ band (C-5), respectively (Hernández-Fernández et al., 2010; Lim et al., 2012). The analysis results of C1s spectrum could verify the amorphous structure of the NC and NMC Analysis high-resolution spectrum of N1s spectra (Figures 3B,D), four peaks around 398, 400.5, 401.6, and 402.8 eV, which can be assigned to pyridinic-N (N-6), pyrrolic-N (N-5), quaternary-N (N-Q), and pyridine-N-oxide (N-X), respectively (Raymundo-Pinero et al., 2002; Long et al., 2008; Braghiroli et al., 2012; Horikawa et al., 2012). Figure 4 shows the different types of nitrogen atoms in a carbon matrix. And the corresponding contents of nitrogen in NC and NMC are shown in Table 2. Obviously, compared with the NMC and NC, the nitrogen chemical state is not change even after CO_2_ activation process. These N-containing functional groups should make both NC and NMC more electrochemically active, indicating it has excellent capacitance properties (Geng et al., 2011; Lim et al., 2012). To sum up, this change of surface chemical compositions is good for electrochemical properties of NMC.

**Table 2:** Nitrogen forms of the NC and NMC.

**Sample**	**Nitrogen forms at.%**			
	**N-6**	**N-5**	**N-Q**	**N-X**
NC	26.1	20.7	46.5	6.7
NMC	22.8	19.4	44.1	13.7

### Electrochemical results

**Figure 5:**
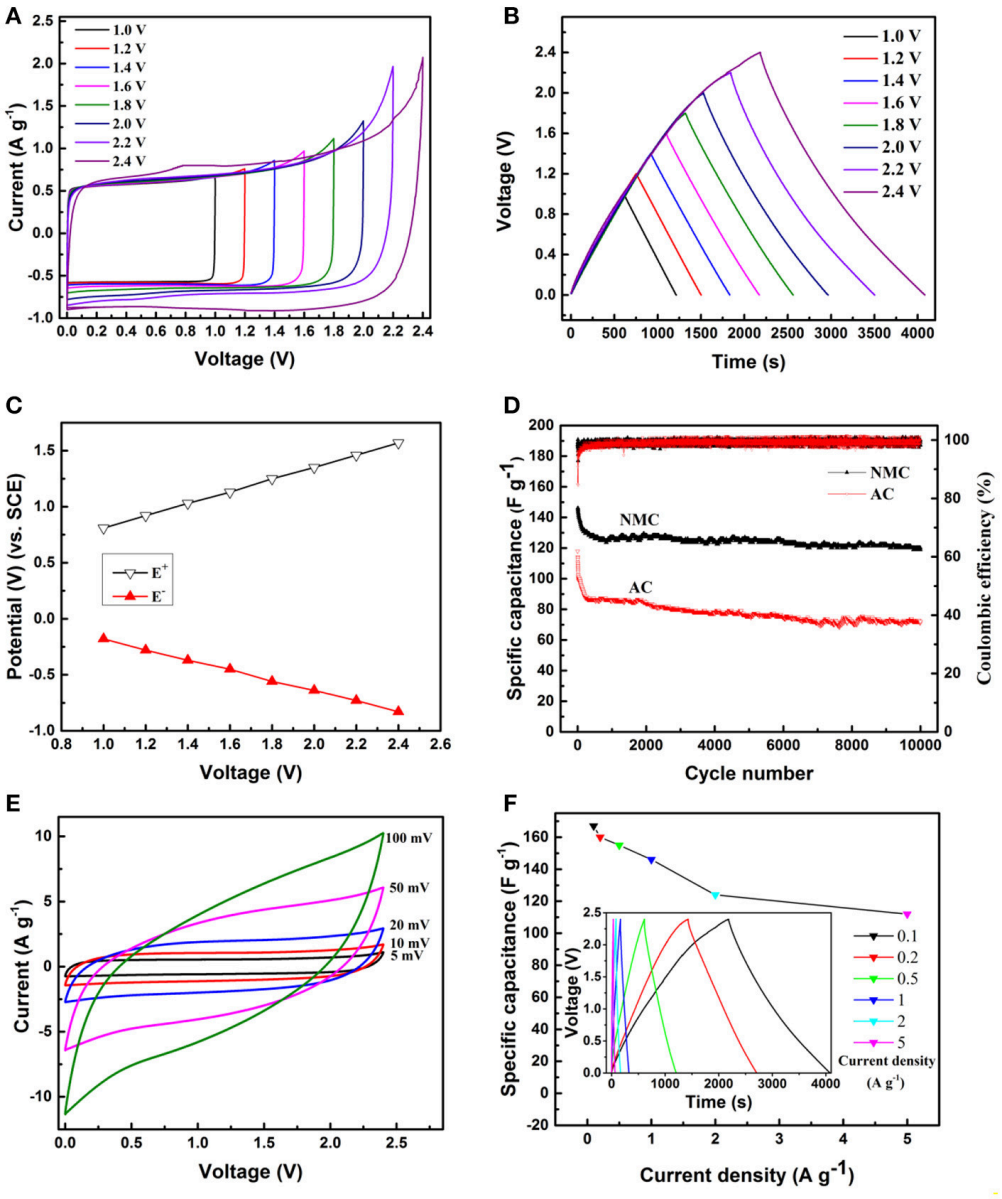
**(A)** CV profiles (5 mV s^−1^) and **(B)** GCD curves (0.1 A g^−1^) of the NMC performed in a symmetric SCs with stepwise shifting of the maximum voltage of 0.2 V. **(C)** Potential changes of the positive (E+) and negative (E–) NMC electrodes. **(D)** Cycle performance of the NMC and AC symmetric SCs at 1 A g^−1^ with a maximum voltage of 2.4 V. **(E)** CV profiles of the NMC SCs at different scan rates. **(F)** GCD curve and capacitance of NMC SCs with different current density. (All above results are tested in 20 m LiTFSI).

The multi-scale porous features of the electrode is expected to facilitate the rapid diffusion of electrolyte ion within the electrode (Qin et al., 2016). To investigate the kinetic stability of NMC in high voltage aqueous SCs, the electrochemical performances were evaluated by using two- and three-electrode cells in 20 m LiTFSI aqueous solution. Figure 5A displays the typical CV curves of the NMC symmetric SCs at the same scan rate of 5 mV s^−1^ in a different operating voltage window from 1 to 2.4 V with stepwise shifting voltage of 0.2 V. It is obvious that LiTFSI-based aqueous electrolytes possess a high stability comparing with organic electrolyte (Zhao et al., 2016), revealing the high adaptability in high voltage cell. Furthermore, the CV profiles of the NMC electrode are nearly rectangle in shape and no obvious redox peaks are detected, which is a very common feature of electrochemical double layer (EDL) capacitor (the schematic of symmetric two-electrode configuration shown in Figure S3). Moreover, it is well known that the charge which is stored within the capacitor may be determined by integrating the CV. The increased area under the curve with increasing scan potential range is clearly observed, indicating an increasing capacitance and high voltage capability of the electrode. This result is consistent with the GCD measurements shown in Figure 5B. The GCD curves of the NMC performed at current density of 0.1 A g^−1^ in a different operating voltage window from 1 to 2.4 V with stepwise shifting voltage of 0.2 V. It can be observed all the GCD curves display a similar linear change of the voltage. With the increasing charge/discharge voltage, the nearly symmetric relationship between the potential vs. time was also observed, suggesting the desired fast charging, and discharging property of the NMC. Slight internal resistance (IR) drops of NMC electrode are observed for any of the curves, which indicate high conductivity of our electrode materials. Calculated from the GCD curves (Figure 5B), the single electrode specific capacitance (C_s_) increase from 120 to 160 F g^−1^ while the operating voltage increase from 1 to 2.4 V. With the working voltage increased, more sufficient surface area can be used to form EDL in the NMC electrode, which mainly attributes to higher Cs. When the symmetric capacitor operate at a high voltage of 2.4 V in 20 m LiTFSI electrolyte, the real potential of the positive and negative electrodes was separately determined by incorporating a SCE reference electrode (Figure 5C). The long term cycling stability applying high voltage of 2.4 V is the critical factor to appraise the practical applications of electrodes. In order to explore this, the cycle stability of NMC was further investigated by repeating the GCD test at a current density of 1 A g^−1^ for 10,000 cycles in 20 m LiTFSI electrolyte as shown in Figure 5D. It was observed that the initial capacitance of NMC electrode is ~150 F g^−1^, and gradually decrease to 120 F g^−1^ during the first 1,000 cycles, but nearly no obvious capacitance decrease during the next thousands cycles. Impressively, even after 10,000 continuous charge/discharge cycles, the NMC electrode retains about 80% of the initial capacitance and exhibits excel lent cycle stability. In contrast, the specific capacitance of AC electrode decreases rapidly from 118 to 86 F g^−1^ during the first 300 cycles, and then gradually decreases to 72 F g^−1^ (only 61% of the initial capacitance is retained) after 10,000 cycles. Figure 5E shows the CV profiles of NMC symmetric SCs at a scan rate of 5, 10, 20, 50, and 100 mV s^−1^, respectively. The rectangular-shape of CV profile is moderately distorted with the increasing scanning rate, which is attributed to the difficult diffusion of ions from electrolyte to the porous structure at high scan rate. In addition, the capacitance decrease with the increasing scan rate, which is consistent with the GCD tests at different current density (Figure 5F). The calculated C_s_ of the NMC in 2.4 V is 167, 160, 155, 146, 124, and 112 F g^−1^ at current densities of 0.1, 0.2, 0.5, 1, 2, and 5 A g^−1^, respectively, demonstrating that the specific capacitance decrease with increasing current density. Furthermore, the retain of capacitance is about 74% when the current density increase from 0.1 to 5 A g^−1^ in 2.4 V.

**Figure 6:**
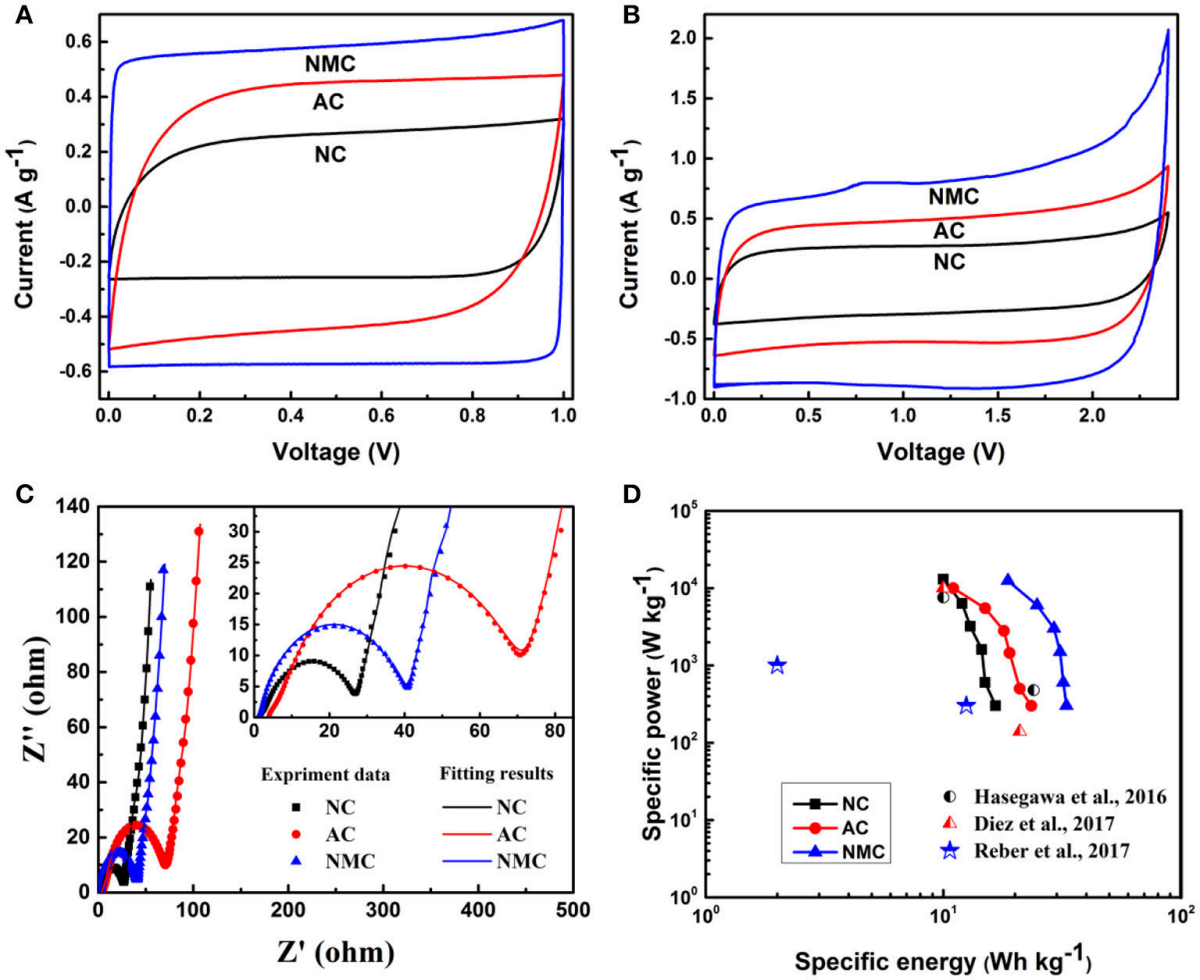
CV profiles of the NC, AC, and NMC in 20 m LiTFSI performed in a symmetric capacitor at 5 mV s^−1^ with the different maximum operating voltage of 1 V **(A)** and 2.4 V **(B)**. **(C)** Nyquist impedance spectra of the NC, AC, and NMC. **(D)** The Ragone plots of the NC, AC, NMC, and relative previous works.

To further discuss the kinetic stability of the NMC under high voltage. The CV and GCD tests for NMC, AC and AC from 1 to 2.4 V were investigated respectively, as shown in Figure 6 and Figure S2. The observed integrated area increases with NC < AC < NMC, indicating that the proper multi-scale pore of NMC is beneficial for the specific capacitance. The Cs of NC, AC, and NMC is 58, 94, and 120 F g^−1^ at the operating potential of 1 V, respectively. Whereas, when the operating potential reaches up to 2.4 V, the Cs increases to 73, 114, and 160 F g^−1^, respectively. The increased Cs is due to the achievement of optimal synergistic effects of multi-scale porous structure and proper nitrogen contents (Zhang et al., 2016). The results obtained here are also in consistent with the SEM morphologies and N_2_ adsorption-desorption test result, suggesting the NM C morphology provides good channels fascinating fast charge intercalation/deintercalation process. As shown in Figure 6C, Nyquist plots for all these three samples consist of a semicircle at high frequency value followed by a slant at low frequency value. The semicircle is ascribed to the charges transfer processes between the electrode and the electrolyte. It is clear that the NC and NMC have the smaller radius of semicircle meaning the smaller charge transfer impedance, which because of the presence of nitrogen provides good electronic conductivity for NC and NMC (Liu et al., 2015). In addition, the slant form Warburg impedance indicates the electrolyte ion diffusion into the electrode. And the AC electrode shows a higher Warburg angel meaning lower ion diffusion which is attributed to the AC have a large number of micropores but lack of micro-channels for the fast ion diffusion (Díez et al., 2017). Figure 6D shows the relationship between energy density and power density of the different electrode materials performed in symmetric capacitor. The specific energy and power were derived from GCD tests at voltage of 2.4 V evaluated using the Equations (1) and (2). It is clear that the symmetric capacitor based on NMC electrodes materials delivered the highest overall energy density of 33 Wh kg^−1^ at 0.3 kW kg^−1^. This result is higher than that of previous homologous works (Gambou-Bosca and Bélanger, 2016; Hasegawa et al., 2016; Díez et al., 2017). Moreover, it is worth noting that the NMC can provide 18 Wh kg^−1^ of specific energy when the power density reaches up to 12 kW kg^−1^. Compared with previous literatures (Hasegawa et al., 2016; Díez et al., 2017; Reber et al., 2017), our works exhibit better performance (Figure 6D) in both energy density and power density.

## Conclusions

N-doped multi-scale porous carbon has been synthesized by a simple sol-gel method and successfully applied in high voltage aqueous electrolyte up to 2.4 V. Morphological and textural characterizations show that the NMC exhibit three dimensional porous channels with a high specific surface area of 3,170 m^2^ g^−1^, large pore volume of 1.880 cm^3^ g^−1^, multi-scale pores and suitable contents of nitrogen functional groups. Electrochemical performances of NMC was observed for a symmetric electrodes system offering good capacitive performance, possessing energy density of 33 Wh kg^−1^ at 0.3 kW kg^−1^ in 20 m LiTFSI electrolyte and stable cycle performance (about 73%) over 10,000 charge-discharge cycles in high voltage of 2.4 V. The high performance characteristics of NMC contribute to the synergistic effect benefiting ion diffusion, transport and adsorption, and charge accumulation. This work demonstrated this nitrogen-doped multi-scale porous carbon is a promising electrode material for high-voltage aqueous electrolyte applications.

## Author contributions

YT developed the concept. XL and CW designed the experiments. XL, RM, and LY conducted the preparation of materials. XL, FY, and RM built the cells and carried out the performance characterizations. ZF and CW supervised the research. XL and FY co-wrote the manuscript. All authors discussed the results and commented on the manuscript.

### Conflict of interest statement

The authors declare that the research was conducted in the absence of any commercial or financial relationships that could be construed as a potential conflict of interest.
