# Supplementary material for: Nitrogen-Doped Multi-Scale Porous Carbon for High Voltage Aqueous Supercapacitors
Source: Front Chem. 2018 Oct 17;6:475. doi: 10.3389/fchem.2018.00475 (PMC6199383; doi:10.3389/fchem.2018.00475)
Supplement: Supplementary file 1 [file Data_Sheet_1.pdf]

frontiers

Supplementary Material

Nitrogen-doped Multi-scaled Porous Carbon for High Voltage Aqueous Supercapacitors

Xichuan Liu[1, 2], Rui Mi[2], Yuan Lei[2], Yang Fan[2], Zhibing Fu[2], Chaoyang Wang[2], Yongjian Tang[1, 2]*

*** Correspondence:** Yongjian Tang**:** tangyongjian2000@sina.com

1 XPS study

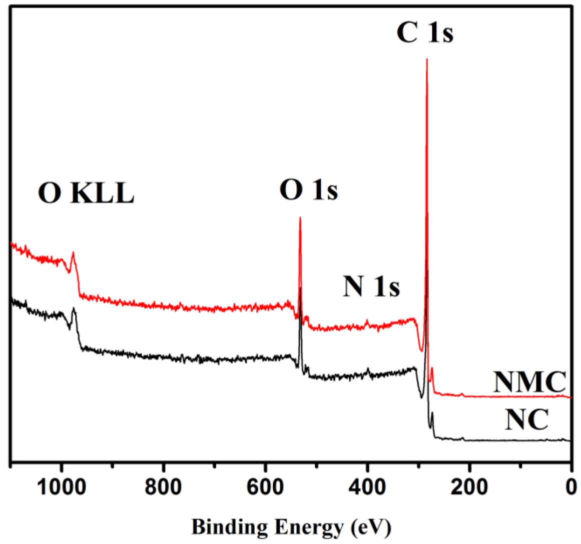

Figure S1. XPS spectrum of the NC and NMC

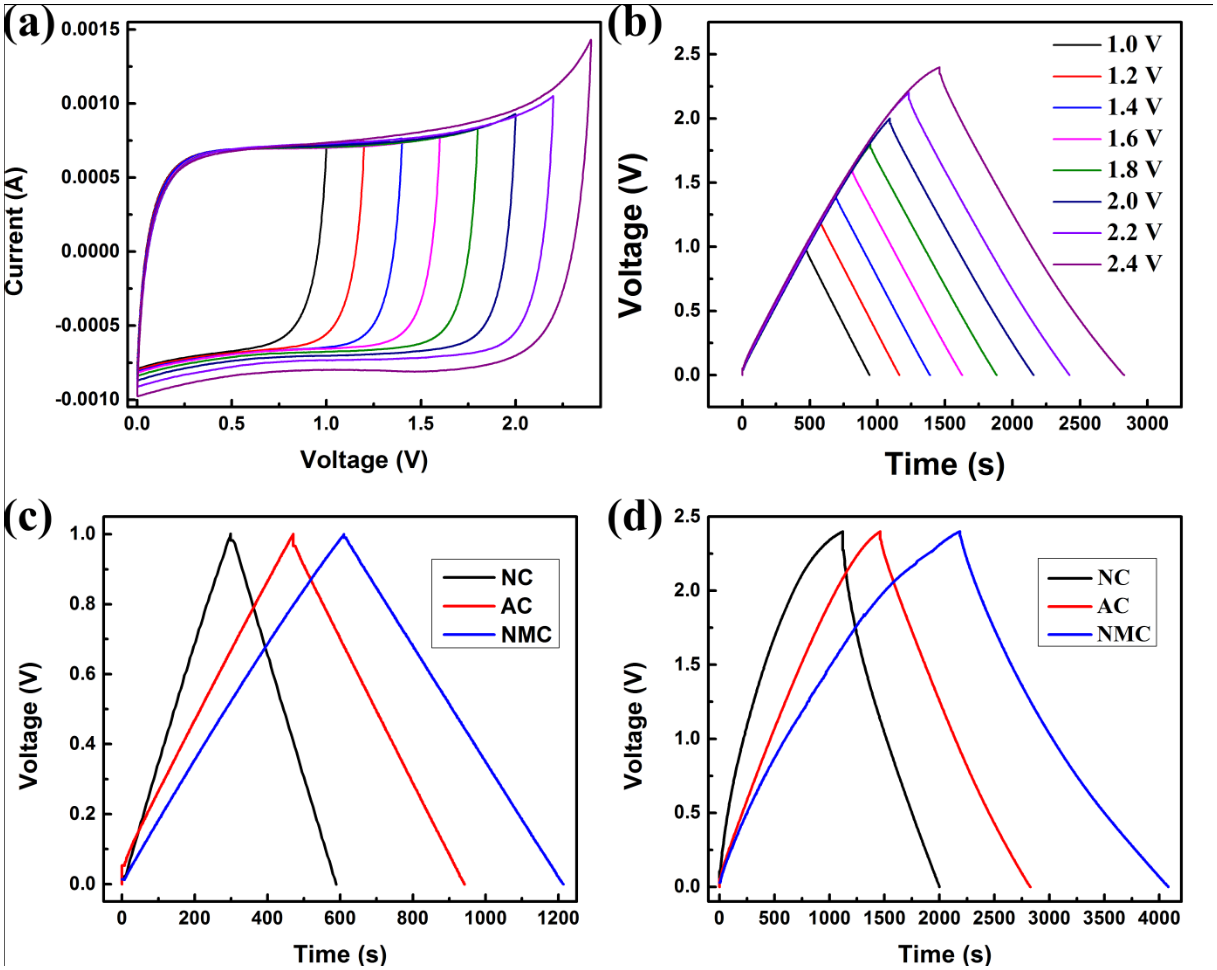

Figure S2. (a) CV profiles (5 mV s^{-1}) and (b) GCD curves (0.1 A g^{-1}) of the AC in 20 m LiTFSI performed in a symmetric capacitor with stepwise shifting of the maximum voltage of 0.2 V. GCD curves of the NC, AC and NMC in 20 m LiTFSI performed in a symmetric capacitor at 5 mV s^{-1} with the different maximum operating voltage of 1 V (c) and 2.4 V (d).

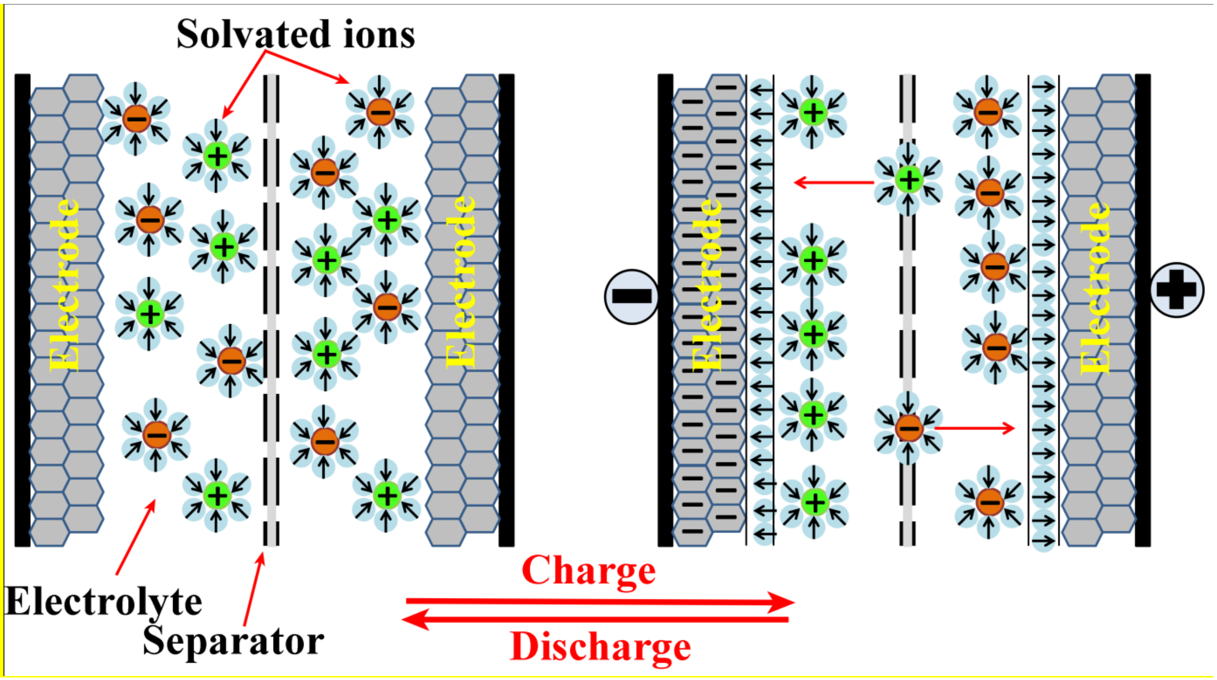

Figure S3. Schematic of symmetric two-electrode configuration
